# Pathology

**Published:** 1913-06

**Authors:** G. Scott Williamson


### PATHOLOGY.

Perhaps the most signal advance made during the past year or so in bacteriology is Noguchi's success in securing a pure **culture of the treponema pallidum** in the test tube.[2] An extract made from such a pure culture has been used as an antigen in the Wassermann technique for the diagnosis of syphilis, and in preparing an extract for use on the lines of von Pirquet's reaction

[2] Noguchi. *J. Exper. Med.*, 1912, xvi. 194.

in tuberculosis. Noguchi further announces the discovery of the treponema pallidum in the brain of a few cases of general paralysis of the insane.[1] Making use of the pallida antigen for comparative study with the original Wassermann antigen prepared from syphilitic liver and other tissues, Craig, Nicholls and Noguchi have demonstrated the superiority of the original antigen in securing a diagnosis in syphilis.[2] In the tertiary stages and after treatment the pallidum antigen appears to give more delicate indication of an actual immunity response. Noguchi claims that in the pallidum antigen we have a means of determining the defensive activity of the host, whereas the Wassermann antigen gives an indication of the infective virility of the active agent, the treponema pallidum.[3]

The value of the **intradermic Luetin reaction** in syphilis remains undetermined. The records[4] of the test indicate a decided specificity, curiously enough almost confined to the tertiary stage of the disease. It is significant that a very large percentage of cases treated with salvarsan and giving a negative Wassermann reaction gave a positive Luetin test.[5] Until the explanation of these findings are clear it would be as well not to claim that a negative Wassermann reaction after treatment indicates freedom from infection.[6] There is much that is obscure and little that is obvious in these diagnostic reactions. That they are all of one type seems undoubted from whatever source the protein employed is derived. There is considerable evidence to associate these reactions with the phenomena of anaphylaxis.[7] Anaphylaxis, according to the authorities, would appear to be a purely physical reaction, probably an absorptive function of a colloidal body.

The **tuberculin reactions** fall into this category. They have been the subject of much study. The general conclusions on the basis of considerable experimental data make it certain that there is little reliance to be placed upon them in clinical diagnosis.[8] It would almost seem that the tuberculin reaction is more an expression of some passive susceptibility rather than any indication of the response of the body to infection by the specific agent. It is certain that these reactions are not a function of the specific toxicity of the organism.[9] Zinsser's

[1] Noguchi and Moore, *Ibid.*, 1913, xvii. 232.

[2] Craig and Nichols, *Ibid.*, 1912, xvi. 336.

[3] Noguchi, *J. Am. M. Ass.*, 1912, lviii. 1163.

[4] Robinson, *J. Cutan. and Genito-Urin. Dis.*, 1912, xxx. 410.

[5] Kämmerer, *München. med. Wchnschr.*, 1912, lix. 1534.

[6] Nobl and Fluss, *Wien. klin. Wchnschr.*, 1912, xxvi. 476.

[7] Belin, *Compt. rend. Soc. de Biol.*, 1912, lxxii. 692.

[8] Wyschelescky, *Ztschr. f. Tuber.*, 1912, xix. 209.

[9] Deycke and Much, *München. Med. Wchnschr.*, 1913, lx. 119.

work on the endotoxins of the typhoid bacillus[1] indicates a separate entity for the fever-producing poisons into which category the tuberculins fall, and casts some doubt on the endotoxic theories associated with that and other organisms. The very meagre justification for the use of tuberculin as a therapeutic agent rests upon this endotoxic hypothesis. It is as well to emphasise this relation between the tuberculin reaction and the condition of anaphylaxis, and to confess our comparative ignorance of the meaning of these phenomena. That anaphylaxis is a dangerous condition is well recognised. A recent observation of Bacmeister's[2] serves to emphasise this point of view. He has demonstrated that virulent tubercle bacilli appear in the blood stream of tuberculous persons after receiving an injection of tuberculin. Blood from four out of fifteen cases served to infect guinea-pigs with acute tuberculosis.

In view of the unreliability of the tuberculin reaction and our ignorance on the subject generally, it would be as well to abandon the use of tuberculin as a diagnostic, and little, if anything, can be said in favour of its use as a therapeutic agent. Its use as a prophylactic against tuberculosis in cattle would seem to be a menace rather than a protection.[3] Griffiths,[4] Weber and others indicate the possibility of actually creating tubercle bacilli carriers as the direct result of prophylactic inoculations of calves. All our experimental findings serve to indicate some extravagance in the claims of the tuberculin advocates. No doubt the neglect of tuberculosis as a subject for research has been in great part due to the satisfaction expressed by those who regard tuberculin as a panacea for the tuberculous.

In a recent issue of the *Journal of Experimental Medicine* Flexner[5] brings forward some further statistics on the use of **anti-meningococcal serum in the treatment of epidemic cerebrospinal fever.** He has collected some 1,300 cases so treated. The results substantiate the earlier reports, and seem to prove conclusively that the serum has a very direct influence on the mortality rate. A reduction of some 20 per cent. must be looked upon with considerable satisfaction. The report deserves very careful study, especially with regard to the effect of the serum in protecting the patients from the rather disastrous sequelæ of the disease. The importance of early diagnosis is

[1] Zinsser, *J. Exper. Med.*, 1913, xvii. 117.

[2] Bacmeister, *München. med. Wchnschr.*, 1913, lx. 343. Laird, *J. Med. Research*, 1912, xxvii. 163. Hammer, *München. med. Wchnschr.*, 1912, lix. 1750.

[3] Weber, *Tuberculose Arbeiten*, 1910.

[4] Griffith, *J. Path. and Bacteriol.*, 1913, xvii. 323.

[5] Flexner, *J. Exper. Med.*, 1913, xvii. 577.

very strikingly emphasised by a table showing the effect upon the mortality rate of early injections of a serum.

| Injected on | No. of cases. | Per cent. died. |
|---|---|---|
| First to third day .. .. | 199 | 18.1 |
| Fourth to seventh day .. | 346 | 27.2 |
| Later than seventh day .. | 666 | 36.5 |

The earliest diagnosis can only be made from an examination of the cerebro-spinal fluid procured by lumbar puncture. Lumbar puncture is a safe and easy procedure, and ought to be resorted to without hesitation in any obscure meningeal condition. Another feature of these results is that they are the result of the employment of inoculation of the anti-serum by dural puncture ; other methods do not yield anything like such good results. Probably it would be as well to turn the disease over to the surgeon for treatment as a septic fever.

As another outcome of the splendid research work of Flexner, **the epidemiology of epidemic poliomyelitis** has been still further elucidated.[1] Both in human and in experimental poliomyelitis the virus has been repeatedly demonstrated in the tonsils, the secretions of the naso-pharyngeal mucosa, and the discharges from the intestine.[2] The marked viability of the virus under adverse conditions, such as drying, low temperature, etc.,[3] and the fact that the virus remains alive in dust,[4] must be considered as making for a fairly complete explanation of the local spread of the disease. Of still further importance is the fact that the virus has been recovered from the naso-pharynx of persons in good health who have been in attendance or contact with acute cases. A very important point to be remembered is that the abortive and cured case can remain a carrier of the virus.[5] It still remains necessary to account for the spread over considerable areas and apart from the possibility of contact infection. Howard and Clark,[6] as the result of their research work, assign some considerable importance to the fact that the domestic fly can carry the virus on its body and in its alimentary tract. This in all probability affords an explanation of the marked seasonal incidence of the

[1] Flexner, *J. Am. M. Ass.*, 1912, lix. 273.
[2] Kling Wernstedt, *Ztschr. f. Immunitätsforsch.*, 1912, xii. 316.
[3] Levaditi and Danulesco, *Compt. rend. Soc. de Biol.*, 1912, lxxii. 543.
[4] Neustädter, *Deutsche Med. Wchnschr.*, 1912, xxxviii. 693.
[5] Landsteiner and Levaditi, *Compt. rend. Acad. d. Sc.*, 1911, clii. 1701.
[6] Howard and Clark, *J. Exper. Med.*, 1912, xvi. 850.

disease. It has also been shown that the blood-sucking insects, the bed bug, is capable of taking the virus with the blood from infected animals and maintaining it in a living state for seven days. This last observation has probably little if anything to do with the spread of the infection in man.

The elaborate researches of Lewis Carrol on **the cultivation of tissue cells in vitro** has started a new line of investigation in cancer research. With cells in more or less pure culture it becomes possible to determine the nature of the stimulus required to initiate mitosis and proliferation. Much interesting work has already been accomplished along these lines with tissue cells corroborating the findings of zoologists experimenting with the ova of fish, etc. The general results seem to indicate that physico-chemical processes depending more or less on the colloidal nature of protein play a considerable part in the process. It does not necessarily follow that the tissues themselves provide the stimulus, so that the possibility of some quite extraneous agent is not excluded. Apart from the question of the etiology of cancerous growth, this work will have immense value in physiological research generally.

G. SCOTT WILLIAMSON.

